# Clinical Lectures on Mental Diseases

**Published:** 1899-02

**Authors:** 


CLINICAL LECTURES ON MENTAL DISEASES. By THOMAS S. CLOUSTON, M. D., Lecturer on Mental Diseases in the University of Edinburgh. New (fifth) edition. Crown 8vo, 750 pages, with nineteen full-page colored plates. Philadelphia and New York: Lea Brothers & Co., Publishers. 1898.

The appearance of a fifth edition of Clouston on mental diseases, shows that the work is fully appreciated in this country as well as in England and needs little introduction to medical readers. As the author rightfully says, the demand for it has absolved its author from any further explanation of its existence. Like the preceding editions it is published in lecture form, twenty in all, embracing the latest researches in general insanity, degeneration and mal-development of the brain. The present edition has been enriched with no less than nineteen full-page colored plates, showing the various pathological conditions of the diseased brain and also of its component parts, microscopic and macroscopic. As an authoritative statement of the best psychiatry, suitable alike for the general physician and the specialist, this volume has won the recognition implied in the demand for five successive editions. W. C. K.

